# Radiological characteristics for guiding intra-arterial therapy in intermediate-stage hepatocellular carcinoma

**DOI:** 10.1186/s12916-025-04446-8

**Published:** 2025-11-06

**Authors:** Chao An, Guanglei Zheng, Jiaoqing Zhou, Ran Wei, Peihong Wu, Mengxuan Zuo

**Affiliations:** 1 Department of Minimal Invasive Intervention, Sun Yat-sen University Cancer Center; State Key Laboratory of Oncology in South China; Collaborative Innovation Center for Cancer Medicine, 651, Dongfeng East Road, Guangzhou, Province Guangdong 510060 P.R. China; 2 Department of Interventional Ultrasound, Fifth Medical Center of Chinese PLA General Hospital, Beijing, P.R. China; 3 Department of International Radiology and Vascular Surgery, The First Affiliated Hospital of Jinan University, Guangzhou, Province Guangdong 510630 P.R. China; 4 Department of Gastrointestinal Surgery, The First Affiliated Hospital of Sun Yat-Sen University, Guangzhou, Province Guangdong 510080 P.R. China

**Keywords:** Hepatocellular carcinoma, Hepatic arterial infusion chemotherapy, Trans-arterial chemoembolization, Radiologic pattern, Propensity score matching

## Abstract

**Background:**

To evaluate and contrast the efficacy and safety of trans-arterial chemoembolization (TACE) versus hepatic arterial infusion chemotherapy (HAIC) for intermediate-stage hepatocellular carcinoma (HCC) based on varying radiologic patterns.

**Methods:**

A retrospective study identified 3,060 consecutive patients with intermediate-stage HCC who underwent initial TACE or HAIC between January 2009 and December 2022. HCC radiological features were categorized into pseudo-capsulated, pseudocapsule breakthrough, confluent multinodular, and infiltrative types. The propensity score matching (PSM) method was employed to minimize selection bias. The progression-free survival (PFS) was compared using the Kaplan–Meier method with the log-rank test. Independent prognostic factors were assessed using a forward stepwise Cox regression model in multivariable analyses.

**Results:**

After propensity score matching, each group in the infiltrative HCC cohort included 237 patients, while the non-infiltrative HCC cohort comprised 262 patients per group. Notably, radiologic patterns were shown with statistical significance in three groups with different tumor burden (*P* < 0.001). Pseudo-capsulated type was dominant (55.6%) in < 6 cohorts, and infiltrative type was dominant (49.5%) in > 12 cohorts. A higher PFS was observed in the HAIC group compared to the TACE group in the infiltrative HCC cohort (*P* < 0.001), but comparable PFS was found between the two groups in the non-infiltrative HCC cohort (*P* = 0.532). In the multivariate analysis, both tumor burden and radiologic morphology showed significant associations with PFS.

**Conclusions:**

HAIC showed favorable outcomes in infiltrative HCC and might warrant further consideration in treatment planning.

**Supplementary Information:**

The online version contains supplementary material available at 10.1186/s12916-025-04446-8.

## Background

Hepatocellular carcinoma (HCC), accounting for 75–85% of liver cancer, is the fourth leading cause of cancer death, with an annual incidence of approximately 840,000 new cases worldwide [1, 2]. In developing countries, regular physical examinations and surveillance remain difficult to promote widely; therefore, many patients are initially diagnosed with large HCC (tumor diameter > 5 cm) [3–6].

International guidelines recommend trans-arterial chemoembolization (TACE) as the first-line treatment for intermediate-stage HCC [4, 7]. Studies have shown that HCC patients with high tumor burden, such as those exceeding seven criteria and unresponsive to conventional TACE (cTACE) using lipiodol mixed with chemotherapeutics, have an objective response rate (ORR) of 16.2–30.3% [8]. Hepatic arterial infusion chemotherapy (HAIC), an intra-arterial therapy (IAT) modality, outperforms intravenous administration by providing sustained high local concentrations of chemotherapeutic agents to the tumor, garnering strong support for treating large HCC [9–11]. The ORR to intra-arterial therapy in intermediate-stage HCC varies considerably, primarily due to the tumor's heterogeneity, which is influenced mainly by the largest tumor diameter.

Tumor burden is crucial in assessing whether HCC patients are suitable candidates for HAIC or TACE. A phase III open-label trial showed that the FOLFOX regimen (oxaliplatin, fluorouracil, and leucovorin) used in HAIC had a higher ORR and a better safety profile compared to cTACE for treating large HCC (diameter > 7 cm) [10]. Furthermore, the radiologic pattern of HCC significantly affects the ORR to IAT [12–15]. The 2022 Barcelona Clinic Liver Cancer (BCLC) guidelines recommend treating infiltrative HCC, characterized by a distinct radiologic pattern, with systemic therapies, such as molecular-targeted therapy, in accordance with the advanced HCC treatment protocol [16]. Previously, our team reported that the ORR and survival outcomes of TACE versus HAIC treatment in pseudo-capsulated and infiltrative HCC, respectively, providing comparative data to guide treatment decisions for two therapeutic modalities of IAT. Yi-Hsiang Huang et al. classified HCC into four subtypes based on radiologic features and found that patients with infiltrative nodular HCC who underwent TACE had the lowest ORR among the subtypes [12].

Given that the FOLFOX regimen of HAIC provides better outcomes, outperforming sorafenib in a previous report [17] and our preliminary exploratory results with a small sample size [18, 19], we hypothesize that HAIC is an effective alternative to TACE for infiltrative HCC. This study compares the survival outcomes and safety of HAIC versus TACE across various radiologic patterns of HCC to contribute evidence informing the updated 2022 BCLC guideline.

## Methods

The Institutional Review Board approved this retrospective study conducted across multiple institutions (IRB: B2022-694–01). The study adhered to the principles of the 1975 Helsinki Declaration, and the need for written informed consent was waived due to its retrospective design. Patient selection, data collection, and analysis followed the STROBE guidelines for observational studies [20]. The data from this study were uploaded to https://www.researchregistry.com/ (ID: researchregistry9425).

### Study design

**Fig. 1:**
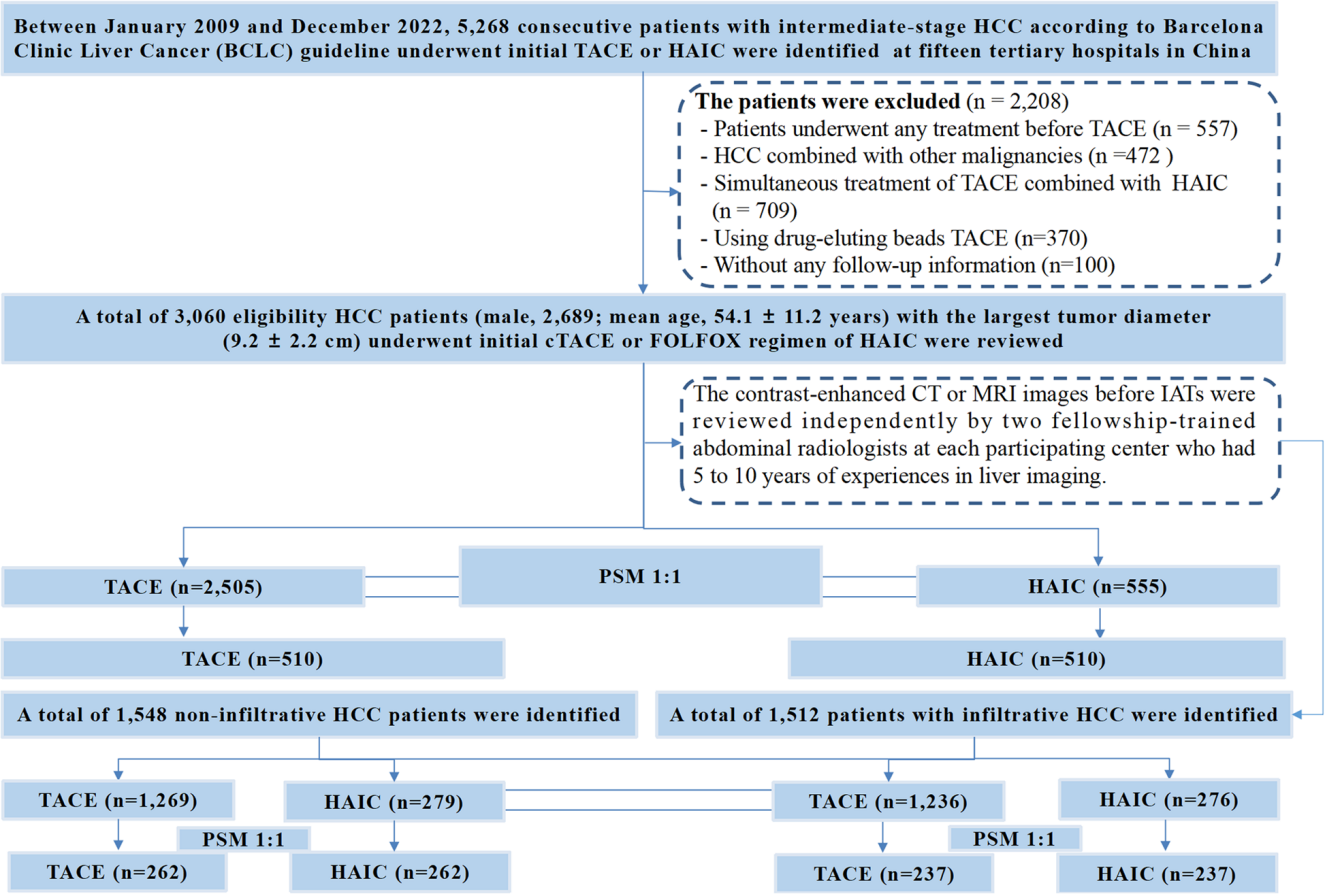
The enrollment pathway of patients with intermediate-stage HCC underwent initial TACE or HAIC according to different radiologic patterns

Between January 2009 and December 2022, 5,268 consecutive patients with intermediate-stage HCC, as defined by the BCLC guideline, received either initial cTACE or the FOLFOX regimen of HAIC. These patients were identified from an in-house medical database, with data source distribution detailed in Additional file 1: Table S1. Inclusion criteria were: (a) age over 18; (b) ECOG performance status of 0 or 1; (c) Child–Pugh class A or B liver function; (d) HCC diagnosis confirmed either pathologically or through AASLD clinical criteria [3]; (e) BCLC B stage verified by liver experts; (f) contrast-enhanced CT or MRI performed within 4 weeks before IAT. Patients were excluded if they: (a) had received previous antitumoral treatment before IAT; (b) had current or past other malignancies besides HCC; (c) had inadequate image quality for assessment; (d) underwent simultaneous TACE and HAIC treatment; (e) received drug-eluting beads TACE (dTACE); or (f) lacked follow-up information. Figure 1 outlines the inclusion and exclusion criteria for patients.

The TACE, HAIC procedures, and criteria for IAT discontinuation are outlined in Additional file 1: E1.1–1.2. The multidisciplinary tumor board selected a therapeutic protocol that includes immune checkpoint inhibitors (ICIs), tyrosine kinase inhibitors (TKIs), and sequential locoregional ablation, based on tumor progression or shrinkage. Details are available in Additional file 1: E1.3–1.4 and Additional file 1: Table S2.

Baseline and pre-IAT clinical data, including demographics, etiologies of chronic liver disease, liver function tests, and laboratory results such as alpha-fetoprotein (AFP), bilirubin, and albumin, were collected. The albumin-bilirubin (ALBI) score, calculated before IAT using the formula (log10 bilirubin [μmol/L] × 0.66) + (albumin [g/L] × -0.085), classifies ALBI grades as follows: grade 1 (≤ -2.60), grade 2 (> -2.60 to -1.39), and grade 3 (> -1.39) [21, 22].

### Image analysis

**Fig. 2:**
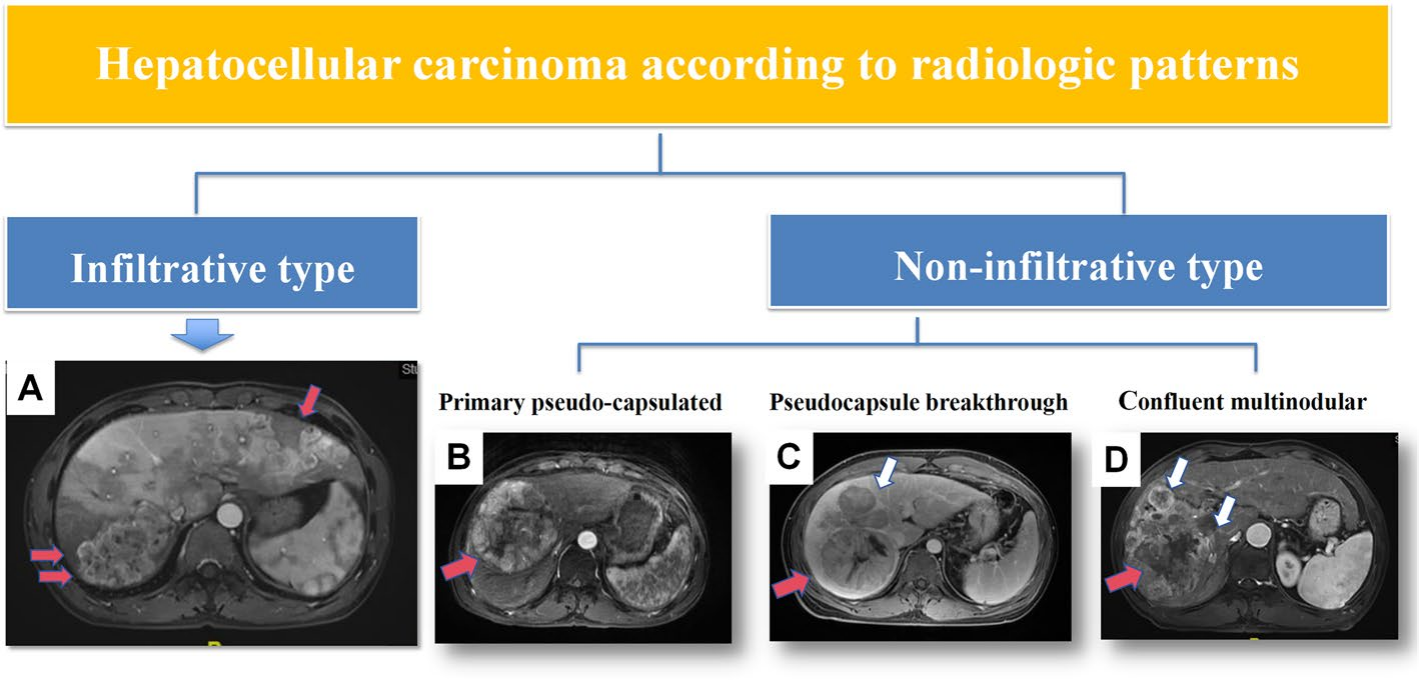
Four types of radiologic features for hepatocellular carcinoma, including pseudo-capsulated type, pseudocapsule breakthrough type, multinodular fusion type, and infiltrative type

The contrast-enhanced CT or MRI images before IAT were reviewed independently by two fellowship-trained abdominal radiologists at each participating center, each with 5–10 years of experience in liver imaging. Reviewers knew all patients had HCC but were blinded to other clinical, histopathologic, therapeutic, and follow-up details. Imaging features were assessed individually for each patient. For patients with multiple tumors, the imaging characteristics of the largest tumor were evaluated. When mixed radiologic patterns were present, the radiologic feature was determined based on the most dominant type. Radiologic features were classified into two categories: non-infiltrative and infiltrative types [23, 24]. Infiltrative HCC often blends with the cirrhotic parenchymal background and lacks demarcation of a discrete mass. Key CT/MRI features diagnostic of infiltrative HCC include: 1) A poorly marginated hepatic lesion; 2) Absence of fibrotic pseudocapsule; 3) Variable (heterogeneous or homogeneous) signal abnormalities; 4) Inhomogeneous contrast washout during portal venous or delayed phases. Additionally, the non-infiltrative type includes pseudo-capsulated, pseudocapsule breakthrough, and confluent multinodular subtypes. A senior radiologist with over 20 years of experience in liver imaging resolved any inter-rater disagreements at each participating center. Figure 2 summarizes the radiologic features of the four HCC types.

### Follow-up and outcomes

HCC patients after IAT underwent regular follow-ups at one month and then every three to six months using serum AFP levels and imaging techniques until death or the final follow-up on October 31, 2023. The primary outcome, based on imaging findings, was classified according to the modified Response Evaluation Criteria in Solid Tumors (mRECIST) [25] into complete response (CR), partial response (PR), stable disease (SD), and progressive disease (PD). ORR is the percentage of CR and PR, while disease control rate (DCR) is the percentage of ORR and SD maintained for more than 4 weeks from initial radiological confirmation. Additional file 1: E1.5 outlines the response criteria for IAT. The secondary endpoints included progression-free survival (PFS) and overall survival (OS). PFS was measured from the start of IAT to the first confirmed tumor recurrence, identified radiologically or histologically as HCC, tumor-in-vein, or distant metastasis according to AASLD criteria, or death from any cause. OS was defined as the duration from the initiation of IAT to death from any cause. Additional file 1: Table S3 shows the post-study treatments administered during follow-up. Additional file 1: Figure S1–2 presents the medical records of patients treated with either cTACE or FOLFOX–HAIC by two radiologic partners. The third outcome involved IAT-related adverse events as defined by the Common Terminology Criteria for Adverse Events version 4.0 [26].

### Statistical analysis

Statistical analysis was performed using SPSS version 23.0 (IBM Corp., NY, USA) and the RMS package in R software version 3.5.3. Inter-rater agreement was assessed with the intraclass correlation coefficient (ICC) for radiologic patterns and Cohen's kappa for binary imaging features. Propensity score matching (PSM) was performed using a 1:1 nearest-neighbor algorithm to balance the groups. Propensity scores from PSM were used to estimate case weights via inverse probability treatment weighting (IPTW) [27]. The weights for the HAIC group were calculated as the inverse of the propensity score, while the weights for the TACE group were calculated as the inverse of one minus the propensity score.

The Kaplan–Meier method, along with the log-rank test, was used to compare cumulative survival. Although a formal sample size calculation was not performed beforehand, the large number of events relative to the variables in the multivariable regression analysis ensured compliance with the 'ten events per variable’ rule, supporting the accuracy of the regression estimates. Univariate and multivariable analyses of independent prognostic factors were conducted using a forward stepwise Cox regression model.

A two-tailed *P* < 0.05 was considered statistically significant.

## Results

### Patients

The study included 3,060 eligible HCC patients (2,689 males; mean age 54.1 ± 11.2 years) with a mean tumor diameter of 9.2 ± 2.2 cm. They were divided into the TACE group (*n* = 2,505) and the HAIC group (*n* = 555). Table 1 presents the baseline characteristics of patients with intermediate-stage HCC, highlighting that the HAIC group exhibited more comorbidities, ascites, higher ALBI scores, and larger tumor diameters compared to the TACE group. Other clinical variables showed no significant differences between the groups. After 1:1 PSM, 510 patients were assigned to each group; no statistically significant differences were found in any variable (Additional file 1: Figure S3). The infiltrative HCC cohort included 237 patients, while the non-infiltrative HCC cohort had 262 patients, as shown in the subgroup analysis. Baseline characteristics are detailed in Table 2. The nearest neighbor method with a 0.1 caliper was used to match these patients (Additional file 1: Figure S4-5).

**Table 1:** Baseline characteristics of the patients with intermediate-stage HCC who received TACE or HAIC

Variables	Unmatched			PSM (1:1)		
	TACE	HAIC	*P*-value	TACE	HAIC	*P*-value
	(*n* = 2505)	(*n* = 555)		(*n* = 510)	(*n* = 510)	
Age (years)			0.216			1.000
≤ 65	2067 (82.51)	445 (80.18)		417 (81.76)	417 (81.76)	
> 65	438 (17.49)	110 (19.82)		93 (18.24)	93 (18.24)	
Gender			1.000			0.703
Female	304 (12.14)	67 (12.07)		60 (11.76)	65 (12.75)	
Male	2201 (87.86)	488 (87.93)		450 (88.24)	445 (87.25)	
ECOG			0.591			0.380
0	2300 (91.82)	514 (92.61)		480 (94.12)	472 (92.55)	
1	205 (8.18)	41 (7.39)		30 (5.88)	38 (7.45)	
Comorbidity			< 0.001			1.000
Absence	2231 (89.06)	450 (81.08)		425 (83.33)	426 (83.53)	
Presence	274 (10.94)	105 (18.92)		85 (16.67)	84 (16.47)	
HBV			0.003			0.392
Absence	128 (5.11)	47 (8.47)		32 (6.27)	40 (7.84)	
Presence	2377 (94.89)	508 (91.53)		478 (93.73)	470 (92.16)	
Ascites			0.020			1.000
Absence	2420 (96.61)	524 (94.41)		484 (94.90)	483 (94.71)	
Presence	85 (3.39)	31 (5.59)		26 (5.10)	27 (5.29)	
Tumor burden			< 0.001			0.830
< 6	835 (33.33)	72 (12.97)		67 (13.14)	72 (14.12)	
6–12	901 (35.97)	142 (25.59)		133 (26.08)	137 (26.86)	
> 12	769 (30.70)	341 (61.44)		310 (60.78)	301 (59.02)	
ALBI grade			< 0.001			0.950
1	1174 (46.87)	307 (55.32)		283 (55.49)	281 (55.10)	
2&3	1331 (53.13)	248 (44.68)		227 (44.51)	229 (44.90)	
AFP, ng/ml			< 0.001			0.661
≤ 400	1440 (57.49)	259 (46.67)		245 (48.04)	237 (46.47)	
> 400	1065 (42.51)	296 (53.33)		265 (51.96)	273 (53.53)	
ALB (ug/ml)^b^	36.5 ± 6.3	36.8 ± 7.2	0.842	36.4 ± 7.7	36.3 ± 7.5	0.887
AST (U/L)^b^	70.2 (59.0, 98.6)	83.47 (52.3,125.3)	< 0.001	76.95 (59.63)	83.30 (129.85)	0.322
ALT (U/L)^b^	57.7 (54.9,112.5)	62.24 (44.6, 85.1)	0.096	59.09 (48.76)	62.69 (67.32)	0.332
TBIL(μmol/L)^b^	19.1 (11.2, 27.5)	17.1 (9.6, 23.6)	0.014	18.3 (8.5, 25.6)	17.2 (11.6, 26.3)	0.313
INR^a^	1.13 ± 0.02	1.09 ± 0.02	0.706	1.09 ± 0.01	1.08 ± 0.02	0.343
PT (s)^a^	12.0 ± 2.51	12.4 ± 4.06	0.011	12.1 ± 0.98	12.2 ± 2.9	0.286
PLT (× 10^9^)^b^	181 (78, 225)	225 (92,311)	0.125	202(107,289)	248 (115,335)	0.187

Data are the number of patients; data in parentheses are percentages unless otherwise indicated

The quantitative data with mean ± standard deviation or median with interquartile range (IQR) were compared by the Mann–Whitney U test

The qualitative data in the two groups were compared by using the chi-square test

The variables matched for PSM included comorbidity, HBV, ascites, ALBI grade, AFP, tumor burden, and AST

A *p*-value < 0.05 suggests statistically significant differences

*Abbreviations*: *HCC* Hepatocellular carcinoma, *HAIC* Hepatic arterial infusion chemotherapy, *TACE* Trans-arterial chemoembolization, *PSM* Propensity score match, *ECOG* Eastern Cooperative Oncology Group, *HBV* Hepatitis type B viral, *AFP* α-fetoprotein, *ALBI* Albumin-bilirubin, *ALB* Albumin, *ALT* Alanine aminotransferase, *AST* Aspartate aminotransferase, *TBIL* Total bilirubin, *PT* Prothrombin time, *INR* International normalized ratio, *PLT* Platelets

^a^mean ± standard deviation

^b^median with interquartile range (IQR)

**Table 2:** Baseline characteristics of the patients with HCC who received TACE or HAIC according to radiological features

Variables	Infiltrative HCC cohort						Non-infiltrative HCC cohort						
	Unmatched			PSM (1:1)			Unmatched				PSM (1:1)		
	TACE (*n* = 1236)	HAIC (*n* = 276)	*P*-value	TACE (*n* = 237)	HAIC (*n* = 237)	*P*-value	TACE (*n* = 1269)	HAIC (*n* = 279)		*P*-value	TACE (*n* = 262)	HAIC (*n* = 262)	*P*-value
Age (years)			0.156			1.000				0.787			0.910
≤ 65	1032 (83.50)	220 (79.71)		194 (81.86)	195 (82.28)		1035 (81.56)		225 (80.65)		215 (82.06)	213 (81.30)	
> 65	204 (16.50)	56 (20.29)		43 (18.14)	42 (17.72)		234 (18.44)		54 (19.35)		47 (17.94)	49 (18.70)	
Sex			0.996			0.763				0.962			0.796
Female	127 (10.28)	29 (10.51)		23 (9.70)	26 (10.97)		177 (13.95)		38 (13.62)		33 (12.60)	36 (13.74)	
Male	1109 (89.72)	247 (89.49)		214 (90.30)	211 (89.03)		1092 (86.05)		241 (86.38)		229 (87.40)	226 (86.26)	
ECOG			0.190			0.234				0.547			0.595
0	1107 (89.56)	255 (92.39)		227 (95.78)	220 (92.83)		1193 (94.01)		259 (92.83)		247 (94.27)	243 (92.75)	
1	129 (10.44)	21 (7.61)		10 (4.22)	17 (7.17)		76 (5.99)		20 (7.17)		15 (5.73)	19 (7.25)	
Comorbidity			0.001			0.899				< 0.001			0.908
Absence	1097 (88.75)	224 (81.16)		199 (83.97)	201 (84.81)		1134 (89.36)		226 (81.00)		216 (82.44)	218 (83.21)	
Presence	139 (11.25)	52 (18.84)		38 (16.03)	36 (15.19)		135 (10.64)		53 (19.00)		46 (17.56)	44 (16.79)	
HBV			0.067			0.279				0.025			1.000
Absence	65 (5.26)	23 (8.33)		13 (5.49)	20 (8.44)		63 (4.96)		24 (8.60)		16 (6.11)	15 (5.73)	
Presence	1171 (94.74)	253 (91.67)		224 (94.51)	217 (91.56)		1206 (95.04)		255 (91.40)		246 (93.89)	247 (94.27)	
Ascites			0.006			0.279				0.855			1.000
Absence	1191 (96.36)	255 (92.39)		217 (91.56)	224 (94.51)		1229 (96.85)		269 (96.42)		252 (96.18)	252 (96.18)	
Presence	45 (3.64)	21 (7.61)		20 (8.44)	13 (5.49)		40 (3.15)		10 (3.58)		10 (3.82)	10 (3.82)	
Tumor burden			< 0.001			0.860				< 0.001			0.874
< 6	390 (31.55)	33 (11.96)		29 (12.24)	33 (13.92)		445 (35.07)		39 (13.98)		39 (14.89)	39 (14.89)	
6–12	466 (37.70)	73 (26.45)		71 (29.96)	69 (29.11)		435 (34.28)		69 (24.73)		63 (24.05)	68 (25.95)	
> 12	380 (30.74)	170 (61.59)		137 (57.81)	135 (56.96)		389 (30.65)		171 (61.29)		160 (61.07)	155 (59.16)	
ALBI grade			< 0.001			0.854				0.139			1.000
1	543 (43.93)	154 (55.80)		128 (54.01)	131 (55.27)		631 (49.72)		153 (54.84)		142 (54.20)	142 (54.20)	
2–3	693 (56.07)	122 (44.20)		109 (45.99)	106 (44.73)		638 (50.28)		126 (45.16)		120 (45.80)	120 (45.80)	
AFP, ng/ml			0.001			0.926				0.002			1.000
≤ 400	671 (54.29)	119 (43.12)		105 (44.30)	103 (43.46)		769 (60.60)		140 (50.18)		132 (50.38)	131 (50.00)	
> 400	565 (45.71)	157 (56.88)		132 (55.70)	134 (56.54)		500 (39.40)		139 (49.82)		130 (49.62)	131 (50.00)	
ALB (ug/ml)^b^	36.2 ± 5.8	36.0 ± 6.6	0.774	36.2 ± 7.7	36.3 ± 7.2	0.902	36.5 ± 6.0		36.8 ± 6.6	0.252	36.4 ± 7.0	36.5 ± 7.2	0.596
AST (U/L)^b^	73.6 (53.5, 103.3)	80.8 (62.8, 113.6)	0.091	81.8 (67.4,105.9)	80.0 (65.2,126.7)	0.771	67.0 (54.2,105.7)		86.0 (57.9,165.16)	0.001	77.1 (50.9,108.6)	86.3 (69.7, 114.9)	0.426
ALT (U/L)^b^	60.4 (37.0, 96.3)	58.4 (44.0,86.3)	0.596	64.2 (39.6,98.6)	58.9 (46.2,112.3)	0.283	55.20 (32.7, 96.8)		65.9 (45.5, 80.5)	0.006	60.15 (35.7,88.2)	66.74 (36.5, 82.4)	0.316
TBIL(μmol/L)^b^	19.5 (14.7, 22.6)	16.0 (10.1, 22.3)	< 0.001	17.0 (9.6,24.6)	15.6 (9.2,23.7)	0.115	18.8 (10.6,21.9)		18.2 (8.5, 23.6)	0.624	17.7 (11.1, 24.3)	18.6 (8.5,29.3)	0.821

Data are the number of patients; data in parentheses are percentages unless otherwise indicated

The quantitative data with mean ± standard deviation or median with interquartile range (IQR) were compared by the Mann–Whitney U test

The qualitative data in the two groups were compared by using the chi-square test

The variables matched for PSM included comorbidity, HBV, ascites, ALBI grade, AFP, tumor burden, and AST

A *p*-value < 0.05 suggests statistically significant differences

*Abbreviations*: *HCC* Hepatocellular carcinoma, *HAIC* Hepatic arterial infusion chemotherapy, *TACE* Trans-arterial chemoembolization, *PSM* Propensity score match, *ECOG* Eastern Cooperative Oncology Group, *HBV* Hepatitis type B viral, *AFP* α-fetoprotein, *ALBI* Albumin-bilirubin, *ALB* Albumin, *ALT* Alanine aminotransferase, *AST* Aspartate aminotransferase, *TBIL* Total bilirubin

^a^mean ± standard deviation

^b^median with interquartile range (IQR)

### Radiologic patterns linked to tumor burden

The correlation between tumor burden (six-to-twelve criteria) and radiologic pattern was assessed based on the classification by Han et al. [28]. Cohen's kappa values were 1.00 (95% confidence interval [CI]: 1,00–1.00) for the pseudo-capsulated type, 0.95 (95% CI: 0.90–0.98) for the pseudocapsule breakthrough type, 0.86 (95% CI: 0.79–0.93) for the multinodular fusion type, and 0.88 (95% CI: 0.81–0.94) for the infiltrative type. A senior radiologist reviewed and resolved the inconsistent results. In patients with a low tumor burden (< 6), the primary pseudo-capsulated type was most common (55.6%), followed by the pseudocapsule breakthrough type (16.7%), the infiltrative type (14.2%), and the multinodular fusion type (13.3%). Conversely, for intermediate tumor burden (6–12), the infiltrative type was dominant (51.1%), followed by the pseudocapsule breakthrough type (20.3%), the multinodular fusion type (18.0%), and the primary pseudo-capsulated type (9.9%). In patients with high tumor burden (> 12), the infiltrative type was most prevalent (49.5%), with the pseudocapsule breakthrough type (23.6%), the multinodular fusion type (21.3%), and the primary pseudo-capsulated type (5.4%).

### Response to the IAT comparison by different radiologic patterns

A higher ORR and DCR to HAIC before and after PSM were observed than TACE in intermediate-stage HCC, as detailed in Additional file 1: Table S4. Post-PSM, the HAIC group showed an ORR of 32.6% and a DCR of 86.4%, while the TACE group had an ORR of 29.5% and a DCR of 72.4%. In the infiltrative HCC cohort, patients in the HAIC group exhibited an ORR of 41.2% and a DCR of 87.2%, compared to the TACE group, which showed an ORR of 26.1% and a DCR of 68.8%. The distribution between the two groups was significantly different (*P* < 0.001). For the non-infiltrative HCC cohort, the TACE group had an ORR of 32.9% and a DCR of 78.6%, while the HAIC group showed an ORR of 35.0% and a DCR of 86.2%. Although the ORR distribution was not significantly different between the groups (*P* = 0.580), HAIC demonstrated a superior DCR compared to TACE (*P* = 0.022).

### Survival outcomes comparison in the total cohort

**Fig. 3:**
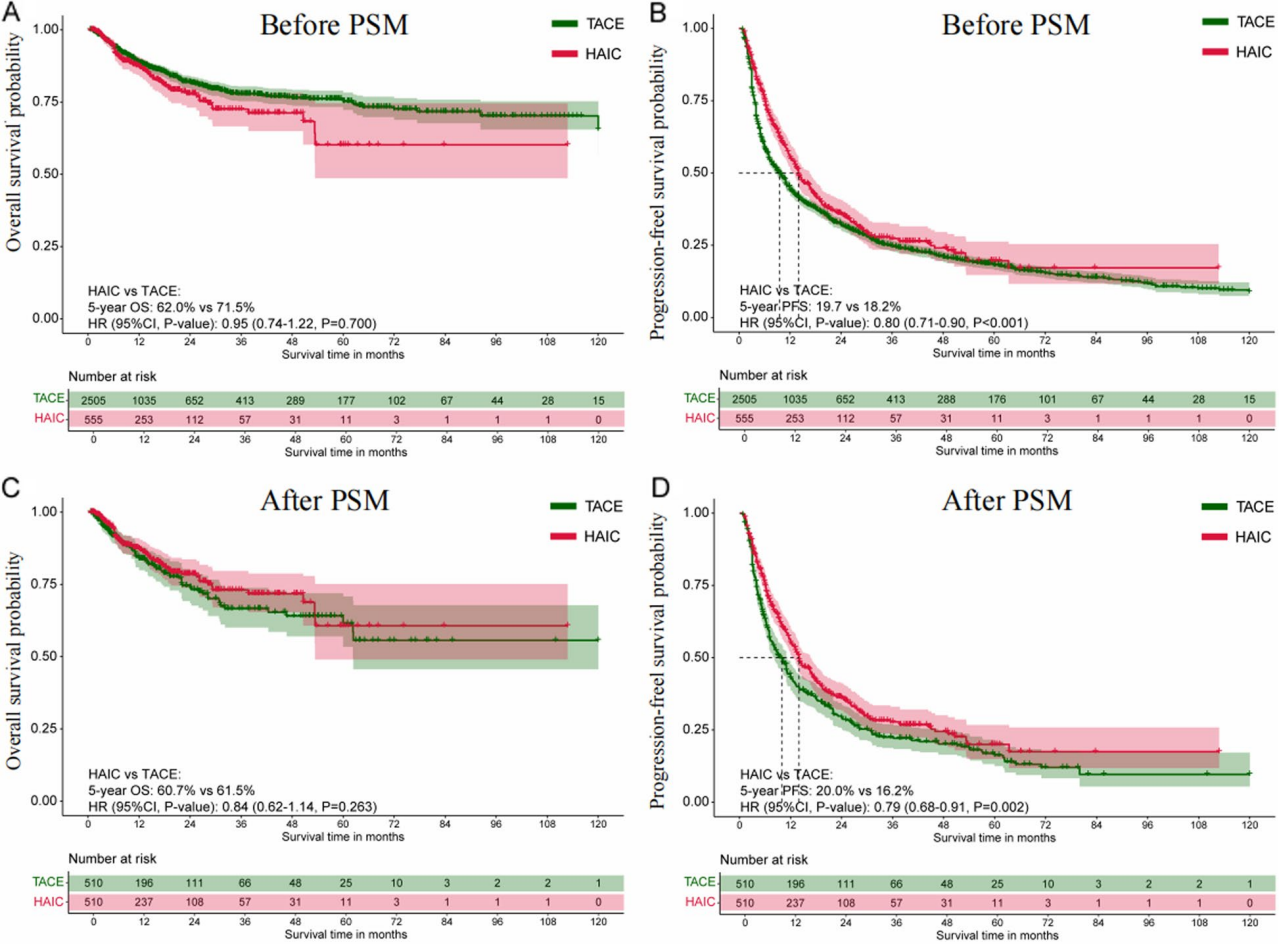
Kaplan–Meier survival curves for patients with intermediate-stage HCC in the total cohort between the HAIC group and the TACE group. **A**-**B** The cumulative OS and PFS comparison before PSM; **C**-**D** The cumulative OS and PFS comparison after PSM

The median follow-up duration was 28.2 months (interquartile range [IQR], 11.5–74.2) for the HAIC group and 29.4 months (IQR, 16.4–72.1) for the TACE group. Kaplan–Meier analysis for the entire cohort showed similar OS between the TACE and HAIC groups, with a 5-year OS rate of 71.5% for TACE and 62.0% for HAIC (hazard ratio [HR] 0.95, 95% CI 0.74–1.22; *P* = 0.700; Fig. 3A). PFS rates were 18.2% for TACE and 19.7% for HAIC, with an HR of 0.80 (95% CI 0.71–0.90; *P* < 0.001; Fig. 3B). The PSM-adjusted Kaplan–Meier analyses showed no significant difference in OS between the two groups, with 5-year OS rates of 60.7% for HAIC and 61.5% for TACE (HR 0.84, 95% CI 0.62–1.14; *P* = 0.263; Fig. 3C). However, the HAIC group had a higher 5-year PFS rate of 20.0% compared to 16.2% in the TACE group (HR, 0.79; 95% CI, 0.68–0.91; *P* = 0.002; Fig. 3D). Additional file 1: Table S5 details the statistical methods used to compare OS and PFS between the two groups. The survival outcome comparisons are consistent with those in the PSM-adjusted cohort.

### Survival outcomes comparison in non-infiltrative and infiltrative HCC cohorts

**Fig. 4:**
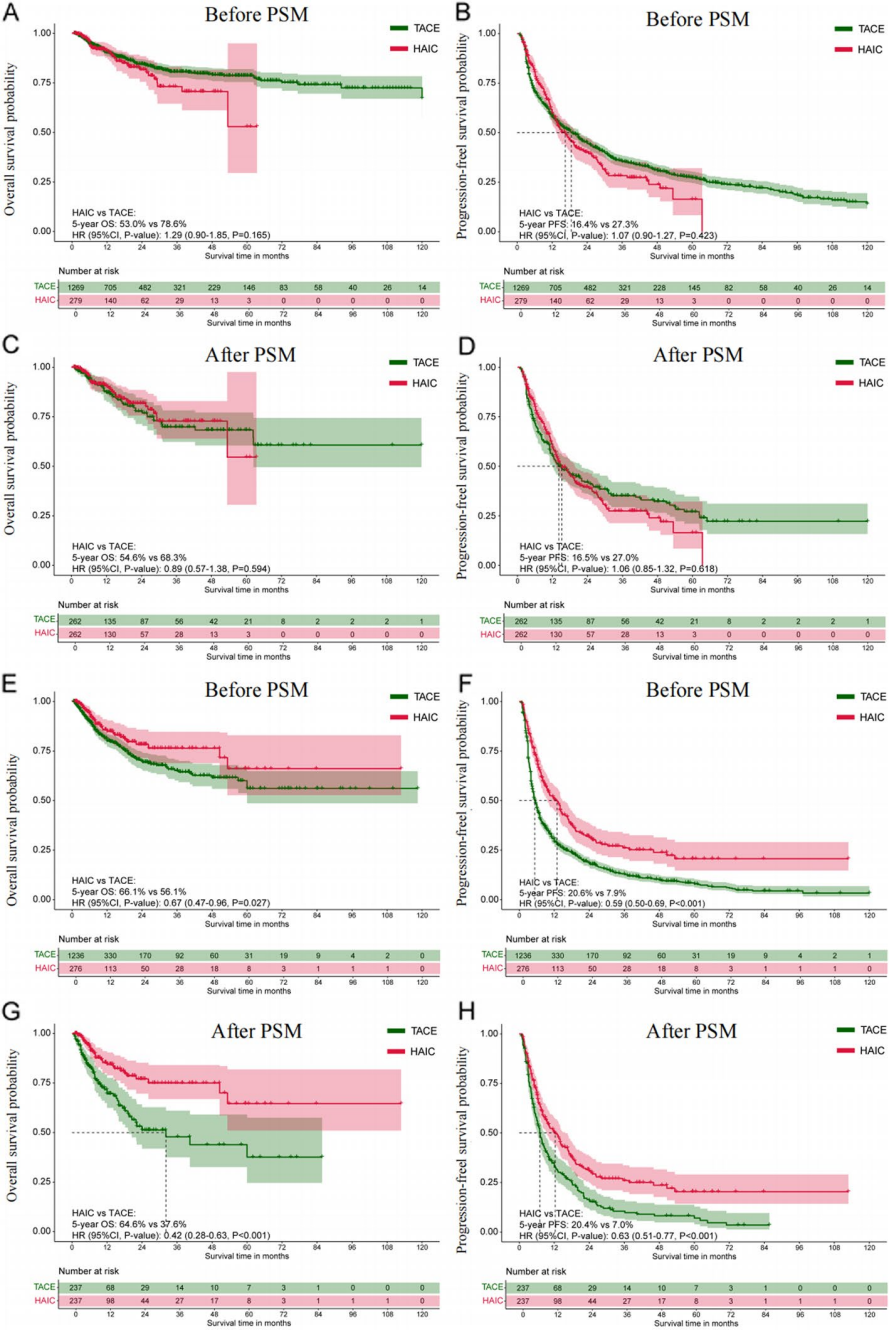
Kaplan–Meier survival curves for patients with HCC in the non-infiltrative and infiltrative HCC cohort between the HAIC group and the TACE group. **A**-**B** The cumulative OS and PFS comparison before PSM in the non-infiltrative cohort; **C**-**D** The cumulative OS and PFS comparison after PSM in the non-infiltrative cohort; **E**–**F** The cumulative OS and PFS comparison before PSM in the infiltrative cohort; **G**-**H** The cumulative OS and PFS comparison after PSM in the infiltrative cohort

The median follow-up duration was 22.5 months (IQR, 10.8–68.5 months) for the HAIC group and 21.9 months (IQR, 13.4–70.8 months) for the TACE group. In the non-infiltrative HCC cohort, Kaplan–Meier analysis showed similar 5-year OS rates (53.0% for HAIC vs. 78.6% for TACE; HR: 1.29, 95% CI: 0.90–1.85; *P* = 0.165) and PFS rates (16.4% for HAIC vs. 27.3% for TACE; HR: 1.07, 95% CI: 0.90–1.27; *P* = 0.423) between the HAIC and TACE groups (Fig. 4A-B). The PSM-adjusted Kaplan–Meier analyses also revealed similar 5-year OS rates between the groups (54.6% vs. 68.3%; HR: 0.89, 95% CI: 0.57–1.38; *P* = 0.594) and comparable 5-year PFS rates (16.5% vs. 27.0%; HR: 1.06, 95% CI: 0.85–1.32; *P* = 0.280), as shown in Fig. 4C-D. Conversely, crude Kaplan–Meier analyses indicated that the infiltrative HCC cohort receiving HAIC had significantly longer cumulative OS and PFS compared to TACE. The 5-year OS rate was 66.1% for HAIC versus 56.1% for TACE (HR: 0.67, 95% CI: 0.46–0.96; *P* = 0.027), and the 5-year PFS rate was 20.6% for HAIC versus 7.9% for TACE (HR: 0.59, 95% CI: 0.50–0.69; *P* < 0.001) (Fig. 4E-F). PSM-adjusted Kaplan–Meier analyses in the infiltrative HCC cohort demonstrated that HAIC was associated with significantly longer cumulative OS and PFS compared to TACE. The 5-year OS rate was 64.6% for HAIC versus 37.6% for TACE (HR: 0.42, 95% CI: 0.28–0.63; *P* < 0.001), and the 5-year PFS rate was 20.4% for HAIC versus 7.0% for TACE (HR: 0.63, 95% CI: 0.51–0.77; *P* < 0.001) (Fig. 4G-H). Additional file 1: Tables S6-7 detail the statistical methods used to compare OS and PFS between groups. The survival outcome comparisons remain consistent with those observed in the PSM-adjusted cohort.

### Factors influencing PFS and OS

In the total cohort, univariate analyses of PFS and OS are presented in Additional file 1: Table S8, and the risk factors with statistical significance were included in the multivariable regression model. In the multivariate analysis (Table 3), tumor burden, AFP, therapeutic modality, and radiologic pattern were independent predictors of PFS. Age, ECOG, ALBI grade, AFP, tumor burden, and radiologic pattern independently predicted OS in the multivariate analysis. In the infiltrative HCC cohort, univariate analyses of PFS and OS were shown in Additional file 1: Table S9. The multivariate analysis identified tumor size, ALBI grade, and therapeutic modality as independent predictors of PFS. Age, AFP, IAT modality, and tumor burden were identified as independent predictors of OS in the multivariate analysis.

**Table 3:** Stepwise multivariable cox analysis for factors associated with PFS and OS

Variables	Total cohorts								Infiltrative HCC cohorts							
	PFS				OS				PFS				OS			
	β	Wald	HR (95% CI)	*P* value	β	Wald	HR (95% CI, *P* value)	*P* value	β	Wald	HR (95% CI, *P* value)	*P* value	β	Wald	HR (95% CI, *P* value)	*P* value
Age (≤ 65, > 65 years)	-0.13	9.03	0.87 (0.78–0.9)	.017	0.10	0.03	1.11 (0.87–1.42)	.386	-0.24	12.08	0.79 (0.67–0.92)	.003	0.29	1.57	1.35 (0.98–1.85)	.063
Gender (Male, Female)	-0.02	0.41	0.98 (0.86–1.11)	.761	-0.15	0.75	0.85 (0.65–1.12)	.260	-0.07	0.01	0.96 (0.80–1.16)	.697	-0.42	4.56	0.66 (0.45–0.95)	.027
ECOG (0, 1)	-0.01	0.02	0.99 (0.84–1.1)	.913	-1.13	13.23	0.32 (0.17–0.61)	< .001	0.15	1.47	1.17 (0.96–1.42)	.112	-1.12	8.55	0.32 (0.14–0.73)	.007
HBV (yes, no)	0.04	1.85	1.04 (0.86–1.2)	.669	0.37	3.96	1.46 (0.87–2.45)	.152	-0.09	0.33	0.91 (0.71–1.17)	.470	0.25	0.40	1.29 (0.66–2.52)	.461
ALBI grade (1, 2–3)	0.32	79.75	1.38 (1.27–1.5)	< .001	0.36	20.27	1.44 (1.19–1.75)	< .001	0.28	31.55	1.32 (1.18–1.49)	< .001	0.40	11.15	1.50 (1.15–1.97)	.003
Turmor buden (≤ 6, > 6)	0.28	103.1	1.33 (1.26–1.4)	< .001	0.44	54.02	1.55 (1.37–1.76)	< .001	0.25	27.21	1.29 (1.20–1.39)	< .001	0.41	19.63	1.51 (1.26–1.80)	< .001
AFP (≤ 400, > 400 ng/ml)	0.25	57.03	1.29 (1.19–1.41)	< .001	0.48	35.96	1.63 (1.34–1.98)	< .001	0.23	12.98	1.26 (1.12–1.42)	< .001	0.52	15.25	1.70 (1.30–2.22)	< .001
Infiltrative HCC (yes, no)	0.65	2.33	1.93 (1.77–2.1)	< .001	0.64	43.27	1.90 (1.56–2.31)	< .001	-	-	-		-	-	-	
IAT modality (TACE, HAIC)	-0.47	13.92	0.62 (0.55–0.7)	< .001	-0.44	0.150	0.64 (0.49–0.83)	.001	-0.69	42.36	0.50 (0.42–0.59)	< .001	-0.73	4.90	0.48 (0.33–0.69)	< .001

*Abbreviations*: *HR* Hazard ratio, *CI* Confidence interval, *IAT* Intra-arterial therapy, *HAIC* Hepatic arterial infusion chemotherapy, *TACE* Trans-arterial chemoembolization, *AFP* Alpha fetoprotein, *ALBI* Albumin-bilirubin, *ECOG* Eastern Cooperative Oncology Group, *OS* Overall survival, *PFS* Progression-free survival

### Safety

The IAT-related adverse events (AEs) comparison between the HAIC group and the TACE group is shown in Table 4. Following PSM 1:1, the occurrence of grade 3–4 adverse events was 20.4% in the HAIC group, similar to the 19.2% observed in the TACE group (*P* = 0.507). The HAIC group experienced more significant abdominal pain in 21.3% of patients (grades 3–4) compared to 12.7% in the TACE group, possibly due to the direct drug effect of oxaliplatin during HAIC. The pain subsided immediately after the oxaliplatin injection was stopped. The frequencies of grade 1–2 AEs, including leukopenia, weight loss, fatigue, diarrhea, vomiting, and elevated creatinine, were comparable between the TACE group and the HAIC group (*P* = 0.982).

**Table 4:** Adverse events comparison between the HAIC group and the TACE group after PSM

Adverse events	HAIC group (*n* = 510)		TACE group (*n* = 510)		*P*-value*	
	Grade 1–2 n (%)	Grade 3–4 n (%)	Grade 1–2 n (%)	Grade 3–4 n (%)	Grade 1–2	Grade 3–4
Patients with at least one event	476 (93.3)	104 (20.4)	484 (94.9)	98 (19.2)	0.982	0.507
Leukopenia	178 (34.9)	69 (13.5)	182 (35.7)	63 (12.3)		
Neutropenia	243 (47.6)	55(10.9)	250 (49.0)	76 (11.3)		
Reduced hemoglobin	58 (11.4)	NA	50 (9.8)	NA		
Coagulation disorder	52 (10.2)	NA	56 (11.0)	NA		
Weight loss	358 (70.2)	14 (2.7)	216 (42.4)	13 (2.5)		
Fever	323 (63.3)	42 (8.2)	368 (72.2)	38 (7.4)		
Fatigue	371 (72.7)	66 (12.9)	381(74.7)	52 (7.7)		
Diarrhea	157 (30.8)	14 (2.7)	68 (6.2)	NA		
Vomiting	367 (72.0)	79 (15.5)	254 (37.7)	65 (12.7)		
HFS	119 (23.4)	12 (2.4)	368 (72.2)	38 (7.5)		
RCCEP	125 (24.5)	16 (3.2)	381(56.7)	52 (10.2)		
Hypothyroidism	14 (2.7)	NA	26 (5.0)	10 (2.0)		
Hypertension	88 (11.3)	NA	68 (12.7)	NA		
Abdominal pain	267 (52.3)	109 (21.3)	254 (49.8)	65 (12.7)		
Elevated ALT	302 (59.2)	137 (26.9)	306 (60.0)	87 (17.1)		
Elevated AST	256 (51.5)	142 (27.8)	243 (47.6)	105 (30.5)		
Elevated TBIL	75 (14.7)	17 (3.3)	79 (11.7)	30 (5.9)		
Elevated creatinine	57 (11.2)	NA	198 (7.4)	NA		
Hypoalbuminemia	299 (74.2)	58 (11.4)	261 (68.5)	48 (9.4)		
Other	70 (13.7)	15 (2.9)	59 (11.6)	16 (3.2)		

*Abbreviations*: *HAIC* Hepatic arterial infusion chemotherapy, *TACE* Trans-arterial chemoembolization, *ALT* Alanine aminotransferase, *AST* Aspartate aminotransferase, *TBIL* Total bilirubin, *HFS* Hand–foot syndrome, *RCCEP* Reactive endothelial proliferation, *NA* No data

*P value < 0.05 suggest statistically significant differences

## Discussion

In 2022, the BCLC guideline revised several sections, mainly including infiltrative HCC as a subtype of intermediate-stage HCC that should be recommended for systematic therapy [16]. However, not all patients with infiltrative hepatocellular carcinoma are eligible for systemic therapy or respond adequately to such treatment. HAIC offers an alternative locoregional therapeutic approach, directly delivering chemotherapeutic drugs into tumors and providing several advantages over traditional chemotherapy, including higher concentration, lower toxicity, and fewer side effects. It has been proven to yield a better ORR and PFS, outperforming sorafenib in a previous report [17]. To date, whether HAIC or TACE, another commonly used IAT, is more suitable for intermediate-stage HCC remains a matter of controversy. Our study aimed to evaluate the antitumor efficacy, long-term survival, and safety of HAIC for infiltrative HCC, offering a potential new treatment option.

We conducted a large-scale study involving patients with intermediate-stage HCC who underwent IAT at 12 hospitals, assessing them based on four radiologic patterns. To reduce selection bias, several analytical methods, including PSM and IPTW, were employed to rigorously determine patient eligibility. In clinical practice, TACE with Yttrium-90 microspheres or drug-eluting beads aimed to standardize the procedure but showed no superiority over cTACE in overall survival [29]. Therefore, we chose cTACE as the control treatment for HAIC in this study. We categorized HCC into four radiological types: pseudo-capsulated, pseudocapsule breakthrough, confluent multinodular, and infiltrative. Our results suggest that the radiologic features are closely associated with tumor burden based on six to twelve criteria. For tumors with a burden of fewer than six, the pseudo-capsulated type is dominant among the four types. Nonetheless, infiltrative HCC was predominant in tumors with a burden greater than six, which aligns with previous studies indicating that it has high invasiveness and increases malignancy with tumor growth [30, 31].

The recent Asia–Pacific Primary Liver Cancer Expert (APPLE) consensus recommends using radiologic features such as confluent multinodular, infiltrative, and extranodular growth types to predict TACE response in intermediate-stage HCC [32]. The study revealed that the TACE group exhibited a lower ORR compared to the HAIC group for infiltrative HCC. Conversely, the non-infiltrative HCC in the TACE group shows a similar ORR to that in the HAIC group. These results suggest that specific radiologic features, such as the infiltrative type, aid in selecting patients for TACE and HAIC in intermediate-stage HCC.

A longer median OS and PFS time was observed in the HAIC group compared to the TACE group for infiltrative HCC, which is consistent with our previous study [19]. However, comparable median PFS and OS times were found for non-infiltrative HCC between the two groups, indicating HAIC can provide a better survival benefit, outperforming TACE in infiltrative HCC rather than non-infiltrative HCC. HAIC is superior to TACE for infiltrative HCC due to several factors: (1) HAIC delivers polonged local high concentrations of chemotherapeutic agents to the tumor for over 24 h; (2) TACE embolization may exacerbate hepatic dysfunction​​ and procedure-related complications. Repeated embolization progressively attenuates tumor vasculature, thereby limiting clinically effective TACE cycles, as most clinical studies cap TACE at 2–4 sessions, whereas HAIC typically permits 4–6 cycles [9, 17, 33, 34]. Furthermore, the large tumor burden and diffuse infiltration pattern​of infiltrative HCC frequently induce collateral circulation or arteriovenous shunting, which impedes complete lipiodol deposition during embolization [23, 35, 36].

Various clinicopathologic variables, including demography and neoplastic characteristics, have a profound impact on PFS and OS in clinical practice. Multivariate analysis identified higher tumor burden, elevated AFP levels, TACE, and infiltrative HCC as independent risk factors for PFS in patients with intermediate-stage HCC. This study examined prognostic factors related to OS, such as age, ECOG performance status, tumor burden, AFP level, ALBI grade, and radiologic features, aligning with findings from prior research [14, 22]. Tumor burden was characterized with six to twelve criteria instead of up to seven criteria. The six-to-twelve criteria represent a novel scoring system for prognostic risk stratification in patients with intermediate-stage HCC undergoing TACE and should be actively promoted [28]. Multivariate analysis indicated that IAT modality did not pose a risk factor for OS in intermediate-stage HCC patients, potentially due to the growing use of TKIs and ICIs following IAT [37, 38]. However, an unexpected opposite result was found in the infiltrative HCC cohort, suggesting that HAIC contributed to improving the survival benefit over TACE.

Adverse events in both groups resembled those observed in a previous phase III trial. In this study, the HAIC group experienced comparable rates of grade 3–4 AEs and hospitalizations compared to the TACE group. Gastrointestinal issues, such as diarrhea and vomiting of any grade, were common in both groups, likely due to the effects of chemotherapeutic drugs. Symptoms like elevated ALT and AST levels and hyperbilirubinemia occurred more frequently in the HAIC group, as fewer sessions of cTACE were performed in clinical practice compared to HAIC. These adverse events were manageable with treatment interruption or dose adjustment but remain challenging to prevent or eliminate entirely. The increased frequency of pyrexia and abdominal pain observed in the HAIC cohort may result from dual mechanisms: direct vascular irritation by oxaliplatin and extensive tumor necrosis associated with higher objective response rates.

Our study had several limitations. First, we only compared the outcomes and antitumor activity of HAIC with TACE for infiltrative HCC, but did not compare HAIC with systemic therapy recommended by the BCLC guideline. Future clinical trials should be designed to compare HAIC with immunotherapy and anti-angiogenic regimens. This combined approach of local interventional therapy integrated with systemic treatment may demonstrate superior efficacy for managing this specific HCC subgroup. Second, patients with HCC who received IAT were enrolled from multiple hospitals; therefore, different treatment protocols and operation techniques may influence the effectiveness of TACE and HAIC. Additionally, a technical factor could also impact the survival outcomes of these two IAT. Third, the leading cause of HCC in China is HBV infection, which differs from the etiologies common in Western countries. It is essential to consider whether this factor affects treatment outcomes. Future efforts should include international multicenter data to validate these findings. Finally, due to the combination of systemic therapy in some patients, the outcome of this study may be affected. We will design clinical trials in the future to further elaborate on the results of this study in order to obtain more reliable results.

## Conclusions

This study serves as a crucial proof-of-concept, providing substantial evidence to validate new therapeutic approaches in the updated BCLC guidelines, and presents clinicians with an alternative to TACE for treating patients with infiltrative HCC. Interventional radiologists must preoperatively assess tumor burden and radiological characteristics to weigh the risk–benefit of HAIC versus TACE.

## Supplementary Information

Additional file 1: Supplementary texts: E1.1-1.5: E1.1 TACE or HAIC procedures. E1.2 Criteria for protocol treatment discontinuation. E1.3 The molecular-targeted agents and immune checkpoint inhibitors protocol. E1.4 Intra-arterial therapy conversion therapy protocol. E 1.5 Assessment criteria of response to intra-arterial therapy. Supplementary tables: Table S1-S9: Table S1. The data source from multiple hospitals in China.Table S2. IAT combination therapy protocol. Table S3. Post-study treatment in the follow-up. Table S4. Associations of tumor response with TACE or HAIC. Table S5. Survival comparison of HAIC versus TACE according to analytic methods in the total cohort. Table S6. Survival comparison of HAIC versus TACE according to analytic methods in the non-infiltrative HCC cohort. Table S7. Survival comparison of HAIC versus TACE according to analytic methods in the infiltrative HCC cohort. Table S8. Prognostic factor analysis for overall survival and disease-free survival in the total cohort. Table S9. Prognostic factor analysis for overall survival and disease-free survival in the infiltrative HCC cohort. Supplementary figures: Figure S1-S5: Figure S1. An example of patients with large pseudo-capsulated HCC receiving HAIC plus oral lenvatinib. Figure S2. An example of patients with complete infiltrative HCC receiving HAIC plus oral lenvatinib. Figure S3 Standardized differences in mean or proportion of variables before and after propensity score matching in the total cohort. Figure S4 Standardized differences in mean or proportion of variables before and after propensity score matching in the non-infiltrative HCC cohort. Figure S5 Standardized differences in mean or proportion of variables before and after propensity score matching in infiltrative HCC cohort.

## Data Availability

The data generated or analyzed in this study can be obtained from the corresponding author upon request.

## Abbreviations

HCC: Hepatocellular carcinoma
TACE: Trans-arterial chemoembolization
HAIC: Hepatic arterial infusion chemotherapy
IAT: Intra-arterial therapy
ORR: Objective response rate
BCLC: Barcelona Clinic Liver Cancer
ICIs: Immune checkpoint inhibitors
TKIs: Tyrosine kinase inhibitors
AFP: Alpha-fetoprotein
ALBI: Albumin-bilirubin
CR: Complete response
PR: Partial response
SD: Stable disease
PD: Progressive disease
DCR: Disease control rate
PFS: Progression-free survival
OS: Overall survival
ICC: Intraclass correlation coefficient
PSM: Propensity score matching
IPTW: Inverse probability treatment weighting
HR: Hazard ratio
CI: Confidence interval
IQR: Interquartile range
AEs: Adverse events
