# Supplementary material for: Radiological characteristics for guiding intra-arterial therapy in intermediate-stage hepatocellular carcinoma
Source: BMC Med. 2025 Nov 6;23:613. doi: 10.1186/s12916-025-04446-8 (PMC12590844; doi:10.1186/s12916-025-04446-8)
Supplement: Supplementary file 1 — Additional file 1: Supplementary texts: E1.1-1.5: E1.1 TACE or HAIC procedures. E1.2 Criteria for protocol treatment discontinuation. E1.3 The molecular-targeted agents and immune checkpoint inhibitors protocol. E1.4 Intra-arterial therapy conversion therapy protocol. E 1.5 Assessment criteria of response to intra-arterial therapy. Supplementary tables: Table S1-S9: Table S1. The data source from multiple hospitals in China.Table S2. IAT combination therapy protocol. Table S3. Post-study treatment in the follow-up. Table S4. Associations of tumor response with TACE or HAIC. Table S5. Survival comparison of HAIC versus TACE according to analytic methods in the total cohort. Table S6. Survival comparison of HAIC versus TACE according to analytic methods in the non-infiltrative HCC cohort. Table S7. Survival comparison of HAIC versus TACE according to analytic methods in the infiltrative HCC cohort. Table S8. Prognostic factor analysis for overall survival and disease-free survival in the total cohort. Table S9. Prognostic factor analysis for overall survival and disease-free survival in the infiltrative HCC cohort. Supplementary figures: Figure S1-S5: Figure S1. An example of patients with large pseudo-capsulated HCC receiving HAIC plus oral lenvatinib. Figure S2. An example of patients with complete infiltrative HCC receiving HAIC plus oral lenvatinib. Figure S3 Standardized differences in mean or proportion of variables before and after propensity score matching in the total cohort. Figure S4 Standardized differences in mean or proportion of variables before and after propensity score matching in the non-infiltrative HCC cohort. Figure S5 Standardized differences in mean or proportion of variables before and after propensity score matching in infiltrative HCC cohort. [file 12916_2025_4446_MOESM1_ESM.doc]

**Radiological Characteristics Determine the Selection of Intra-arterial Therapy Modality for Intermediate-Stage Hepatocellular Carcinoma**

**These supplementary materials include:**

- **Supplementary texts: E1.1-1.5**

**E1.1** TACE or HAIC procedures.

**E1.2**  Criteria for protocol treatment discontinuation.

**E1.3** The molecular-targeted agents and immune checkpoint inhibitors protocol

**E1.4** Intra-arterial therapy conversion therapy protocol.

**E 1.5** Assessment criteria of response to intra-arterial therapy.

- **Supplementary tables: Table S1-S9**

**Table S1**. The data source from multiple hospitals in China.

**Table S2**. IAT combination therapy protocol

**Table S3.** Post-study treatment in the follow-up.

**Table S4.** Associations of tumor response with TACE or HAIC.

**Table S5.** Survival comparison of HAIC versus TACE according to analytic methods in the total cohort

**Table S6.** Survival comparison of HAIC versus TACE according to analytic methods in the non-infiltrative HCC cohort

**Table S7.** Survival comparison of HAIC versus TACE according to analytic methods in the infiltrative HCC cohort

**Table S8.** Prognostic factor analysis for overall survival and disease-free survival in the total cohort.

**Table S9.** Prognostic factor analysis for overall survival and disease-free survival in the infiltrative HCC cohort

- **Supplementary figures: Figure S1-S5**

**Figure S1.** An example of patients with large pseudo-capsulated HCC receiving HAIC plus oral lenvatinib. (A) The pre-HAIC enhanced images, including A1. DSA shows the tumor with diffuse distribution and abundant arterial blood supply. A2-3. Enhanced contrast MRI shows the tumor with diffuse distribution in the arterial and portal phase and typical fast-forward, fast-out presence. After four cycles of HAIC, the large HCC shrank significantly, and then one month after HAIC treatment, the tumor thrombus disappeared with oral lenvatinib.

**Figure S2.** An example of patients with complete infiltrative HCC receiving HAIC plus oral lenvatinib. (A) The pre-HAIC enhanced images, including A1. DSA shows the tumor with diffuse distribution and abundant arterial blood supply. A2-3. Enhanced contrast MRI shows the tumor with diffuse distribution in the arterial and portal phases, and a typical fast-forward, fast-out presence. After four cycles of HAIC, the large HCC shrank significantly, and then one month after HAIC treatment, the tumor thrombus disappeared with oral lenvatinib.

**Figure S3** Standardized differences in mean or proportion of variables before and after propensity score matching in the total cohort

**Figure S4** Standardized differences in mean or proportion of variables before and after propensity score matching in the non-infiltrative HCC cohort

**Figure S5** Standardized differences in mean or proportion of variables before and after propensity score matching in infiltrative HCC cohort

**1. Supplementary tests**

**E1.1 TACE or HAIC procedures.**

Transarterial chemoembolization (TACE) or hepatic arterial infusion chemotherapy (HAIC) procedures have been described in our previous report[1-4]. All equipment of IAT procedures included i) digital subtraction angiography (Philips, type FD 20 1250 mA, Amsterdam, Netherlands); ii) the artery sheath catheter was inserted into the femoral artery using the modified Seldinger technique; iii) A 5-Fr Yashiro catheter (Terumo, Tokyo, Japan) was advanced into the celiac trunk and superior mesenteric artery to assess the feeding hepatic artery; iv) A 2.7-Fr micro-catheter (Terumo, Tokyo, Japan) was inserted in the feeding artery. The therapeutic principles of IAT procedures were as follows: 1) TACE: the feeding artery was selected or super-selected whenever possible. Emulsion, which consisted of 10–20 ml Lipiodol, 30–50 mg platinum drugs, and 20–40 mg epirubicin, was injected slowly until the offending vessel occluded. If necessary, embolization using gel foam mixed with contrast medium was performed to reduce the residual blood flow until there was no longer any tumor staining after repeat angiography. 2) HAIC: all chemo-drugs were given by HAIC through the micro-catheter. A modified FOLFOX6 regimen, including oxaliplatin (130 mg/m^2^ infusion for 3 h on day 1), leucovorin (200 mg/m^2^ for 3–5 hours on day 1), and Fluorouracil (400 mg/m^2^ in bolus, and then 2,400 mg/m^2^ continuous infusion 23-46 h) was applied. Treatment was repeated every 3 weeks and commonly 4–6 cycles unless intrahepatic lesions progressed or toxicity became unacceptable.

**E1.2 Criteria for protocol treatment discontinuation**

1. Tumor progression. Dynamic CT or MRI assessed the progression of disease (PD) based on modified Response Evaluation Criteria in Solid Tumor (mRECIST).
2. Intolerable adverse event

i) Patient cannot resume IATs after 30 days of interruption due to an adverse event;

ii) An adverse event that meets the criteria for chemotherapy agent dose reduction occurs after the dose was already reduced to the lowest level;

iii) Life-threatening adverse event;

C) The need for another anticancer treatment due to downstaging at the physician’s discretion;

D) IATs become technically infeasible;

E) Inadequate blood or bone marrow (leukopenia count < 3.0×10^9^/L, platelet count < 50×10^9^/L, and hemoglobin < 8.0 g/L)

C. Patient requests to discontinue the study；

D.Death.

**E1.3 The molecular-targeted agents and immune checkpoint inhibitors protocol**

During HAIC or TACE treatment, molecular-targeted agents (MTAs) and immune checkpoint inhibitors (ICIs) were used to control the intrahepatic and extrahepatic progression. Oral first-line targeted chemotherapy, including sorafenib and lenvatinib, was started 1–5 days after the first HAIC or TACE session and continually administered. Once the disease progresses or 3-4 AEs occur, the second-line treatment regimen (regorafenib or apatinib) can be administered. Oral lenvatinib (Lenvimafi; Eisai Co., Ltd.) was administered to the patients with Ad-HCCs. The initial dose was determined based on the patient’s body weight and liver function. Patients weighing > 60 kg with the Child–Pugh A classification started at a dose of 12 mg once daily. Patients weighing < 60 kg with the same liver function began at a dose of 8 mg once daily. A reduction in dosage or interruption of treatment was implemented when AEs were detected. Lenvatinib was administered unless patients were intolerant of radiological tumor progression or AEs. ICI immunotherapy was performed after 1-3 days of IAT treatment and every 3 weeks intravenously. Fixed-dose administration of PD-1 was used until disease progression or unexpected toxicity. The dose and interval of TKIs allowed changes depending on toxicity and disease conditions [5].

**E1.4 Intra-arterial therapy conversion therapy protocol**

The target tumors were down-staged to BCLC-A stage from BCLC-B/C stages, and the tumor burden was reduced to meet the Milan criterion after multiple cycles of TACE or HAIC treatment [6]. The down-staged HCC patients underwent treatment methods including surgical resection, imaging-guided thermal ablation, and SBRT. Among them, a resectable tumor was defined as the complete removal of all macroscopic tumor tissue and an expected remnant liver volume of no less than 250 mL/M.

**E 1.5 Assessment criteria of response to intra-arterial therapy**

Before the TACE or HAIC treatment was discontinued, HCC patients’ follow-up was performed every 2-4 weeks after each treatment session. The first response to HAIC or TACE was defined as the response assessment according to the modified RECIST (mRECIST) guidelines by local investigators after the first HAIC or TACE treatment. The optimal response to HAIC or TACE was defined as the response assessment after at least two cycles of HAIC or TACE. All responses had to be assessed by the same imaging method of baseline per modified Response Evaluation Criteria in Solid Tumors (mRECIST) criteria. Two radiologists (reader 1, H.R., with 10 years of experience in abdominal imaging, and reader 2, C.A., with 8 years of experience in abdominal imaging), blinded to clinical procedures, were chiefly responsible for the assessment. To minimize interpretation bias, the evaluation must achieve a final consensus between two radiologists. In the process of HAIC or TACE treatment, the most significant reduction in tumor diameter and number is considered the optimal treatment response. However, if new tumors appear or the number of tumors increases, even if the largest tumor shrinks significantly after treatment, it is also considered tumor progression.

**References**

1. Li QJ, He MK, Chen HW, et al. Hepatic Arterial Infusion of Oxaliplatin, Fluorouracil, and Leucovorin Versus Transarterial Chemoembolization for Large Hepatocellular Carcinoma: A Randomized Phase III Trial. J Clin Oncol 2022;40:150-60.
2. Li SH, Mei J, Cheng Y, et al. Postoperative Adjuvant Hepatic Arterial Infusion Chemotherapy With FOLFOX in Hepatocellular Carcinoma With Microvascular Invasion: A Multicenter, Phase III, Randomized Study. J Clin Oncol 2023;41:1898-908.
3. Zhu K, Huang J, Lai L et al. Medium or Large Hepatocellular Carcinoma: Sorafenib Combined with Transarterial Chemoembolization and Radiofrequency Ablation. Radiology 288:300-307
4. An C, Yao W, Zuo M, Li W, Chen Q, Wu P. Pseudo-capsulated Hepatocellular Carcinoma: Hepatic Arterial Infusion Chemotherapy Versus Transcatheter Arterial Chemoembolization. Acad Radiol 2024;31:833-43.
5. Shimose S, Iwamoto H, Tanaka M, et al. Alternating Lenvatinib and Trans-Arterial Therapy Prolongs Overall Survival in Patients with Inter-Mediate Stage HepatoCellular Carcinoma: A Propensity Score Matching Study. Cancers (Basel) 2021;13
6. Shi F, Wu M, Lian SS et al. Radiofrequency Ablation Following Downstaging of Hepatocellular Carcinoma by Using Transarterial Chemoembolization: Long-term Outcomes. Radiology 293:707-715

**2. Supplementary tables**

**Table S1.** The data source from multi-center hospitals.

| **Hospitals** | **HAIC group**  **n=555** | **TACE group**  **n=2505** |
| --- | --- | --- |
| **Southern China, n (%)** | | |
| Sun Yat-sen University Cancer Center | 423 (76.3) | 1383 (55.2) |
| The First Affiliated Hospital of Sun Yat-sen University | 53 (9.5) | 190 (7.6) |
| The Third Affiliated Hospital of Sun Yat-sen University | 40 (7.2) | 220 (8.8) |
| Guangdong Provincial People's Hospital | 22 (4.0) | 110 (4.4) |
| Jinan University First Affiliated Hospital | 21 (1.5) | 159 (6.3) |
| Guangzhou Cancer Hospital | 4 (0.7) | 162 (6.5) |
| Affiliated Hospital of Southern Medical University | 0 (0) | 50 (2.0) |
| The First Affiliated Hospital of Nanchang University | 0 (0) | 21 (0.8) |
| **Northern China, n (%)** | | |
| The First Affiliated Hospital of Peking University | 0 (0) | 23 (0.9) |
| Cancer Hospital, Chinese Academy of Medical Sciences, and Peking Union Medical College | 3 (0.6) | 162 (6.5) |
| Luhe Hospital, Capital Medical University | 1 (0.1) | 18 (0.7) |
| Chinese PLA General Hospital | 0 (0) | 7 (0.3) |
| **Abbreviation:** HAIC, hepatic arterial infusion chemotherapy; TACE, transarterial chemoembolization. | | |

**Table S2.** IAT's combination therapy protocol.

| **Combination therapy protocol** | **TACE combination therapy**  **(n = 581)** | **HAIC combination**  **therapy**  **(n =377)** |
| --- | --- | --- |
| **TKIs, n (%)** | | |
| Sorafenib | 110 (18.9) | 92 (24.4) |
| Lenvatinib | 299 (51.5) | 88 (23.3) |
| Regorafenib | 72 (12.4) | 36 (9.5) |
| Apatinib | 45 (7.7) | 42 (11.1) |
| Sorafenib plus Lenvatinib | 42 (7.2) | 10 (2.6) |
| Sorafenib plus Regorafenib | 37 (6.4) | 11 (2.9) |
| Lenvatinib plus Regorafenib | 21 (3.6) | 55 (14.6) |
| **ICIs, n (%)** | | |
| Camrelizumab | 100 (17.2) | 87 (23.1) |
| Tislelizumab | 79 (13.6) | 12 (3.2) |
| Stintilimab | 112 (19.3) | 54 (14.3) |
| Others | 15 (2.6) | 8 (2.1) |
| TKIs plus ICIs, n (%) | 154 (26.5) | 112 (29.7) |
| **Local treatments, n (%)** | | |
| Surgical resection | 212 (36.5) | 96 (25.5) |
| MWA | 137 (23.6) | 189 (50.1) |
| RFA | 23 (4.0) | 16 (4.2) |
| SBRT | 45 (7.7) | 4 (1.0) |
| Others | 5 (0.9) | 1 (0.3) |
| **Abbreviation**: HAIC, hepatic arterial infusion chemotherapy; IAT, ; ICIs, immune checkpoint inhibitors; TKIs, tyrosine kinase inhibitors; MWA, microwave ablation; RFA, radiofrequency ablation; SBRT, stereotactic body radiation therapy. | | |

**Table S3.** Post-study treatment in follow-up.

| **Treatment** | **HAIC group**  **(n=555)** (%) | **TACE group**  **(n=2,505)** (%) | **P-Value** |
| --- | --- | --- | --- |
| Number of patients with at least one treatment after disease downstaging, for maintaining the response of the study treatment, or due to the intolerable toxicity |  |  | 0.002 |
| Absence | 376 (67.7) | 1824 (72.8) |  |
| Presence | 179 (32.3) | 681(27.2) |  |
| Cancer disappeared | 38 (2.8) | 68 (2.7) |  |
| Thermal ablation | 56(4.1) | 318(12.6) |  |
| Surgical resection | 124(9.0) | 62 (2.2) |  |
| Radiotherapy for vascular invasion | 45(3.3) | 54 (1.6) |  |
| TACE or HAIC | 47(3.4) | 56 (1.7) |  |
| TKIs | 195 (35.2) | 562 (22.3) |  |
| ICIs | 149 (26.8) | 256 (10.2) |  |
| TKIs plus ICIs | 84(15.5) | 215 (8.6) |  |
| Number of patients with at least one 2-line treatment after disease progression | 77 (13.9) | 378 (25.5) | <0.001 |
| Thermal ablation | 4 (0.7) | 12(0.8) |  |
| Surgical resection | 5 (0.8) | 28(0.5) |  |
| Radiotherapy for vascular invasion | 6 (1.1) | 26(0.4) |  |
| TACE or HAIC | 21(3.8) | 16(1.0) |  |
| TKI | 42 (7.6) | 36(2.4) |  |
| ICI | 6 (1.1) | 14(0.9) |  |
| Number of patients with at least one 3-line treatment after disease progression | 112(20.1) | 481(19.2) | <0.001 |
| Thermal ablation | 6(1.1) | 15 (0.9) |  |
| Surgical resection | 0 (0) | 0 (0) |  |
| Radiotherapy for vascular invasion | 2(0.3) | 13(0.5) |  |
| TACE | 5(0.8) | 13(0.5) |  |
| TKIs | 17(3.1) | 11(0.4) |  |
| ICIs | 5(0.8) | 28(1.1) |  |
| TKIs plus ICIs | 4(0.7) | 10 (0.4) |  |
| TACE, transarterial chemoembolization; HAIC, hepatic arterial infusion chemotherapy; ICIs, immune checkpoint inhibitors; TKIs, tyrosine kinase inhibitors; MWA, microwave ablation; RFA, radiofrequency ablation; SBRT, stereotactic body radiation therapy | | | |

| **Table S4.** Associations of tumor response with TACE or HAIC. | | | | | | | | | | | |
| --- | --- | --- | --- | --- | --- | --- | --- | --- | --- | --- | --- |
| **Variables** | **Case No.** | | **HAIC vs cTACE (unadjusted)** | | | **HAIC vs cTACE (PSM*)** | | | **HAIC vs cTACE (IPTW*)** | | |
|  | **HAIC (n=555)** | **TACE (n=2505)** | **OR** | **95% CI** | **P-value** | **OR** | **95% CI** | **P-value** | **OR** | **95% CI** | **P-value** |
| **Tumor response after 1st IAT** | | | | | | | | | | | |
| PDR (%) | 13.2 | 25.4 | ref. |  |  | ref. |  |  | ref. |  |  |
| CRR (%) | 0.4 | 2.0 | 0.42 | 0.17, 1.03 | 0.058 | 0.76 | 0.20, 2.88 | 0.690 | 0.69 | 0.37, 1.31 | 0.782 |
| PRR (%) | 25.2 | 25.1 | 1.93 | 0.85, 2.43 | 0.859 | 3.07 | 0.56, 2.38 | 0.925 | 2.64 | 0.52, 3.13 | 0.911 |
| SDR (%) | 60.1 | 47.5 | 2.43 | 0.98, 2.98 | 0.067 | 2.85 | 0.72, 3.69 | 0.785 | 2.35 | 0.75, 2.71 | 0.563 |
| ORR (%) | 25.6 | 27.1 | 1.82 | 0.82, 2.28 | 0.578 | 2.99 | 0.66, 4.24 | 0.907 | 2.51 | 0.68, 2.97 | 0.856 |
| DCR (%) | 86.8 | 74.6 | 2.21 | 1.82, 2.68 | <0.001 | 2.92 | 0.92, 2.68 | 0.105 | 2.92 | 0.97, 3.76 | 0.098 |
| **Optimal tumor response after multi-cycle IATs** | | | | | | | | | | | |
| PDR (%) | 13.6 | 21.6 | ref. |  |  | ref. |  |  | ref. |  |  |
| CRR (%) | 0.7 | 2.7 | 0.50 | 0.24, 1.01 | 0.054 | 1.05 | 0.36, 3.08 | 0.93 | 0.75 | 0.45, 1.25 | 0.260 |
| PRR (%) | 31.9 | 26.4 | 2.23 | 1.79, 2.79 | <0.001 | 3.28 | 2.34, 4.60 | <0.001 | 2.56 | 2.17, 3.00 | <0.001 |
| SDR (%) | 53.6 | 48.2 | 2.23 | 1.82, 2.73 | <0.001 | 2.68 | 2.08, 3.45 | <0.001 | 2.06 | 1.78, 2.37 | <0.001 |
| ORR (%) | 32.6 | 26.1 | 2.07 | 1.66, 2.58 | <0.001 | 3.19 | 2.28, 4.45 | <0.001 | 2.41 | 2.06, 2.83 | <0.001 |
| DCR (%) | 86.2 | 78.4 | 2.17 | 1.79, 2.63 | <0.001 | 2.86 | 2.23, 3.65 | <0.001 | 2.17 | 1.89, 2.49 | <0.001 |

| **Abbreviation:** HAIC, hepatic arterial infusion chemotherapy; TACE, transarterial chemoembolization; CRR, complete response rate; PRR, partial response rate; SDR, stable disease rate; PDR, progression disease rate; ORR, objective response rate; DCR, disease control rate. |
| --- |

| **Table S5.** Survival comparison of HAIC versus TACE according to analytic methods in total cohort | | | |
| --- | --- | --- | --- |
| **Analysis** | **HR^*^** | **95% CI** | **P-value** |
| **OS†** |  |  |  |
| Adjusted† | 0.64 | 0.49-0.83 | 0.001 |
| IPTW | 0.73 | 0.54-0.99 | 0.047 |
| PSM | 0.84 | 0.62-1.14 | 0.263 |
| **PFS**‡ |  |  |  |
| Adjusted‡ | 0.62 | 0.55-0.70 | 0.001 |
| IPTW | 0.65 | 0.56-0.75 | <0.001 |
| PSM | 0.79 | 0.68-0.91 | 0.002 |
| **Note.—*** HRs for the TACE group compared with the HAIC group.  † Adjusted for age at diagnosis, HCC burden, and ALBI grade;  ‡ Adjusted for age at diagnosis and , HCC diameter;  **Abbreviations:** HAIC, hepatic arterial infusion chemotherapy; TACE, transarterial chemoembolization; IPTW, inverse probability treatment weighting; PSM, propensity score matching; HR, hazard ratio; CI, confidence interval; OS: overall survival; DFS: disease-free survival. | | | |

| **Table S6**. Survival comparison of HAIC versus TACE according to analytic methods in non-infiltrative cohort | | | |
| --- | --- | --- | --- |
| **Analysis** | **HR^*^** | **95% CI** | **P-value** |
| **OS†** |  |  |  |
| Adjusted† | 0.93 | 0.64-1.36 | 0.725 |
| IPTW | 0.97 | 0.63-1.49 | 0.916 |
| PSM | 0.89 | 0.57-1.38 | 0.594 |
| **PFS**‡ |  |  |  |
| Adjusted‡ | 0.87 | 0.73-1.04 | 0.120 |
| IPTW | 0.87 | 0.71-1.08 | 0.207 |
| PSM | 1.06 | 0.85-1.32 | 0.618 |
| **Note.—*** HRs for the TACE group compared with the HAIC group.  † Adjusted for age at diagnosis, AFP, tumor burden, and ALBI grade;  ‡ Adjusted for tumor burden;  **Abbreviations:**HAIC, hepatic arterial infusion chemotherapy; TACE, transarterial chemoembolization; IPTW, inverse probability treatment weighting; PSM, propensity score matching; HR, hazard ratio; CI, confidence interval; OS: overall survival; DFS:disease-free survival. | | | |

| **Table S7**. Survival comparison of HAIC versus TACE according to analytic methods in infiltrative cohort | | | |
| --- | --- | --- | --- |
| **Analysis** | **HR^*^** | **95% CI** | **P-value** |
| **OS†** |  |  |  |
| Adjusted† | 0.48 | 0.33-0.69 | <0.001 |
| IPTW | 0.52 | 0.33-0.79 | 0.002 |
| PSM | 0.42 | 0.28-0.63 | <0.001 |
| **PFS**‡ |  |  |  |
| Adjusted‡ | 0.50 | 0.42-0.59 | 0.001 |
| IPTW | 0.49 | 0.40-0.61 | <0.001 |
| PSM | 0.63 | 0.51-0.77 | <0.001 |
| **Note.—*** HRs for the TACE group compared with the HAIC group.  † Adjusted for AFP, tumor burden, and ALBI grade;  ‡ Adjusted for AFP and , tumor burden;  **Abbreviations:** HAIC, hepatic arterial infusion chemotherapy; TACE, transarterial chemoembolization; IPTW, inverse probability treatment weighting; PSM, propensity score matching; HR, hazard ratio; CI, confidence interval; OS: overall survival; DFS:disease-free survival. | | | |

**Table S8**. Prognostic factor analysis for overall survival and disease-free survival in total cohort.

| **Variables** | **Disease-free survival** | | **Overall survival** | |
| --- | --- | --- | --- | --- |
|  | **HR (95% CI)** | **P-value** | **HR (95% CI)** | **P-value** |
| Age (years), ≥65 | 1.15 (1.02-1.33) | 0.032 | 0.78 (0.52-1.67) | 0.525 |
| Gender, female | 0.78 (0.62-1.34) | 0.475 | 0.82 (0.54-1.88) | 0.325 |
| ECOG PS, 0 | 0.45 (0.22-1.22) | 0.287 | 1.02 (0.89-1.16) | 0.797 |
| HBV, presence | 1.42 (0.75-2.46) | 0.563 | 1.14 (0.94-1.38) | 0.184 |
| Comorbidities, presence | 0.88 (0.62-1.26) | 0.424 | 0.91 (0.79-1.04) | 0.155 |
| Infiltrative HCC, absence | 3.56 (2.23-5.12) | < 0.001 | 0.92 (0.74-1.15) | 0.480 |
| Tumor burden, > 6 | 2.33 (1.27-4.89) | < 0.001 | 1.18 (1.12-1.25) | < 0.001 |
| AFP (ng/mL), >400 | 1.72 (1.32-2.59) | < 0.001 | 1.32 (1.22-1.44) | < 0.001 |
| ALBI grade, 2-3 | 1.52 (1.05-1.85) | < 0.001 | 1.27 (1.17-1.38) | < 0.001 |
| Treatment modality, HAIC | 0.55 (0.34-0.89) | < 0.001 | 0.80 (0.71-0.90) | < 0.001 |
| **Abbreviations:** ECOG, Eastern Cooperative Oncology Group; HBV, hepatitis type B viral; AFP, α-fetoprotein; ALBI, albumin-bilirubin; HAIC, hepatic arterial infusion chemotherapy. | | | | |

**Table S9**. Prognostic factor analysis for overall survival and disease-free survival in infiltrative HCC cohort.

| **Variables** | **Disease-free survival** | | **Overall survival** | |
| --- | --- | --- | --- | --- |
|  | **HR (95% CI)** | **P-value** | **HR (95% CI)** | **P-value** |
| Age (years), ≥65 | 1.15 (1.02-1.33) | 0.032 | 0.78 (0.52-1.67) | 0.525 |
| Gender, female | 0.78 (0.62-1.34) | 0.475 | 0.82 (0.54-1.88) | 0.325 |
| ECOG PS, 0 | 0.45 (0.22-1.22) | 0.287 | 1.02 (0.89-1.16) | 0.797 |
| HBV, presence | 1.42 (0.75-2.46) | 0.563 | 1.14 (0.94-1.38) | 0.184 |
| Comorbidities, presence | 0.88 (0.62-1.26) | 0.424 | 0.91 (0.79-1.04) | 0.155 |
| Infiltrative HCC, absence | 3.56 (2.23-5.12) | < 0.001 | 0.92 (0.74-1.15) | 0.480 |
| Tumor burden, > 6 | 2.33 (1.27-4.89) | < 0.001 | 1.18 (1.12-1.25) | < 0.001 |
| AFP (ng/mL), >400 | 1.72 (1.32-2.59) | < 0.001 | 1.32 (1.22-1.44) | < 0.001 |
| ALBI grade, 2-3 | 1.52 (1.05-1.85) | < 0.001 | 1.27 (1.17-1.38) | < 0.001 |
| Treatment modality, HAIC | 0.55 (0.34-0.89) | < 0.001 | 0.80 (0.71-0.90) | < 0.001 |
| **Abbreviations:** ECOG, Eastern Cooperative Oncology Group; HBV, hepatitis type B viral; AFP, α-fetoprotein; ALBI, albumin-bilirubin; HAIC, hepatic arterial infusion chemotherapy. | | | | |

**3. Supplementary figures**

**Figure S1**


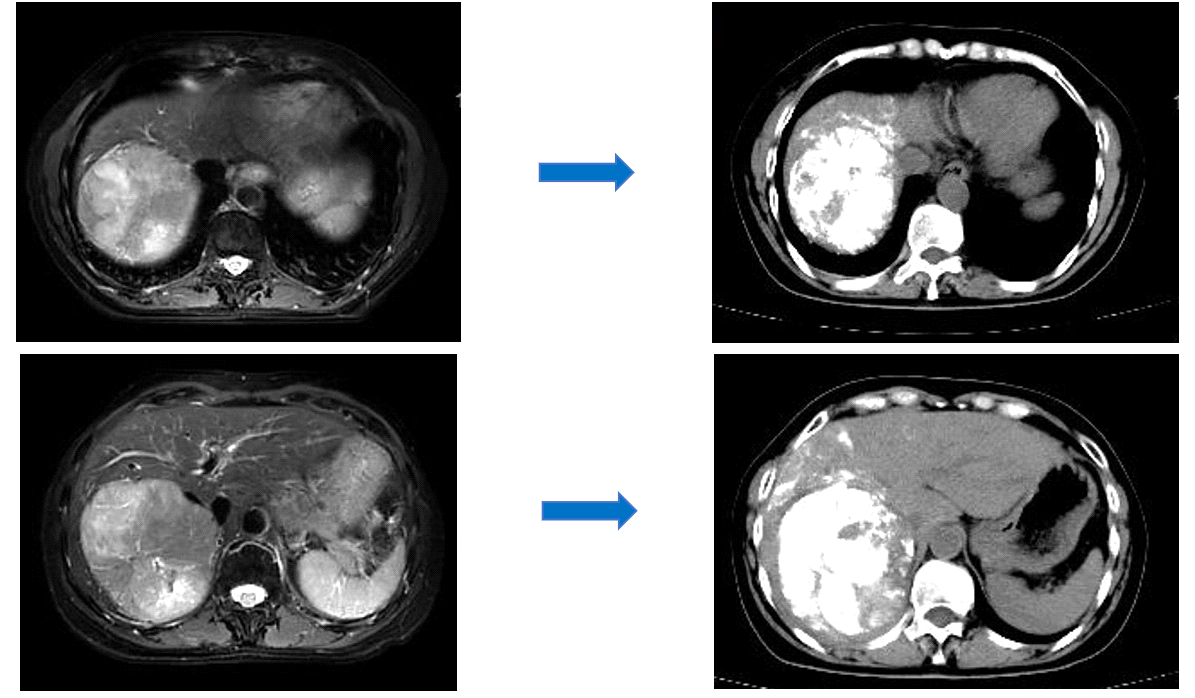


**Figure S1** An example of patients with large pseudo-capsulated HCC receiving HAIC plus oral lenvatinib. (A) the pre-HAIC enhanced images including A1. DSA show the tumor with diffuse distribution and abundant arterial blood supply；A2-3. Enhanced contrast MRI show the tumor with diffuse distribution in arterial and portal phase and typical fast-forward, fast-out presence. After four cycles of HAIC, the large HCC shrank significantly, and then one month after HAIC treatment, the tumor thrombus disappeared with oral lenvatinib.

**Figure S2**


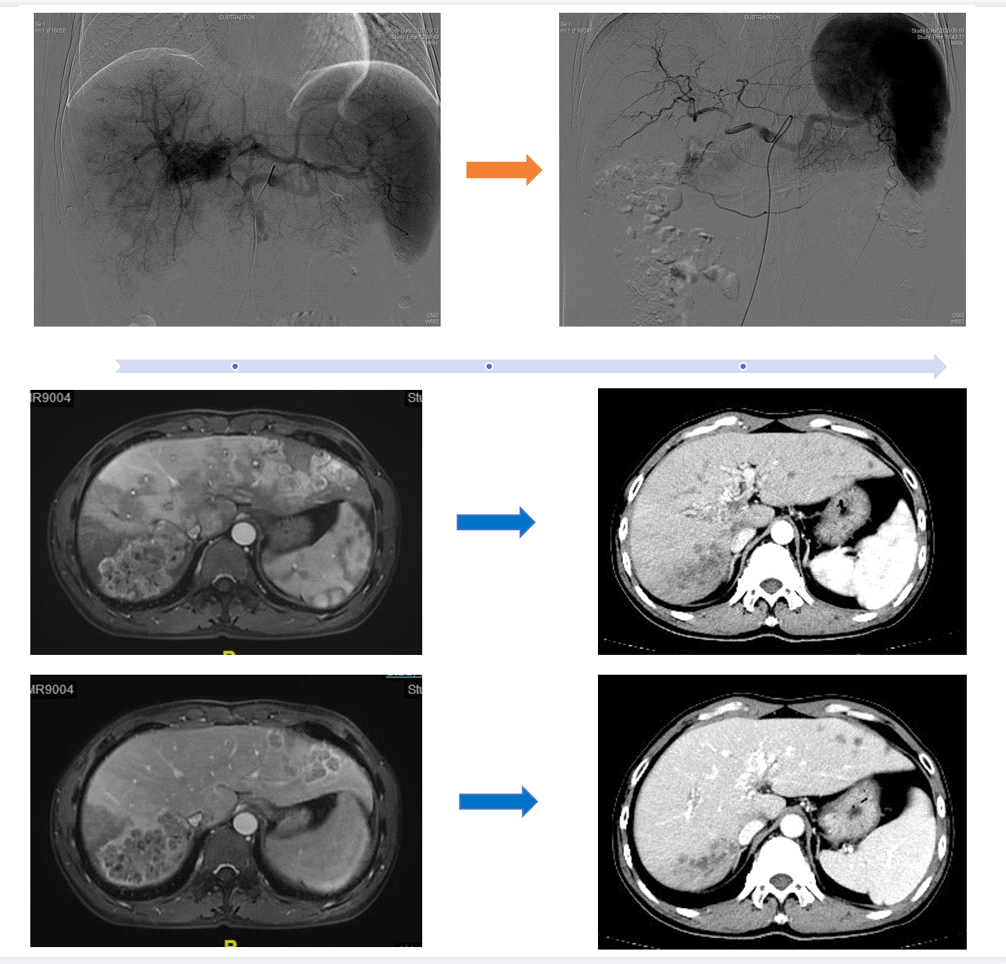
**Figure S2** An example of patients with complete infiltrative HCC receiving HAIC plus oral lenvatinib. (A) the pre-HAIC enhanced images including A1. DSA show the tumor with diffuse distribution and abundant arterial blood supply；A2-3. Enhanced contrast MRI show the tumor with diffuse distribution in arterial and portal phase and typical fast-forward, fast-out presence. After four cycles of HAIC, the large HCC shrank significantly, and then one month after HAIC treatment, the tumor thrombus disappeared with oral lenvatib.

**Figure S3**


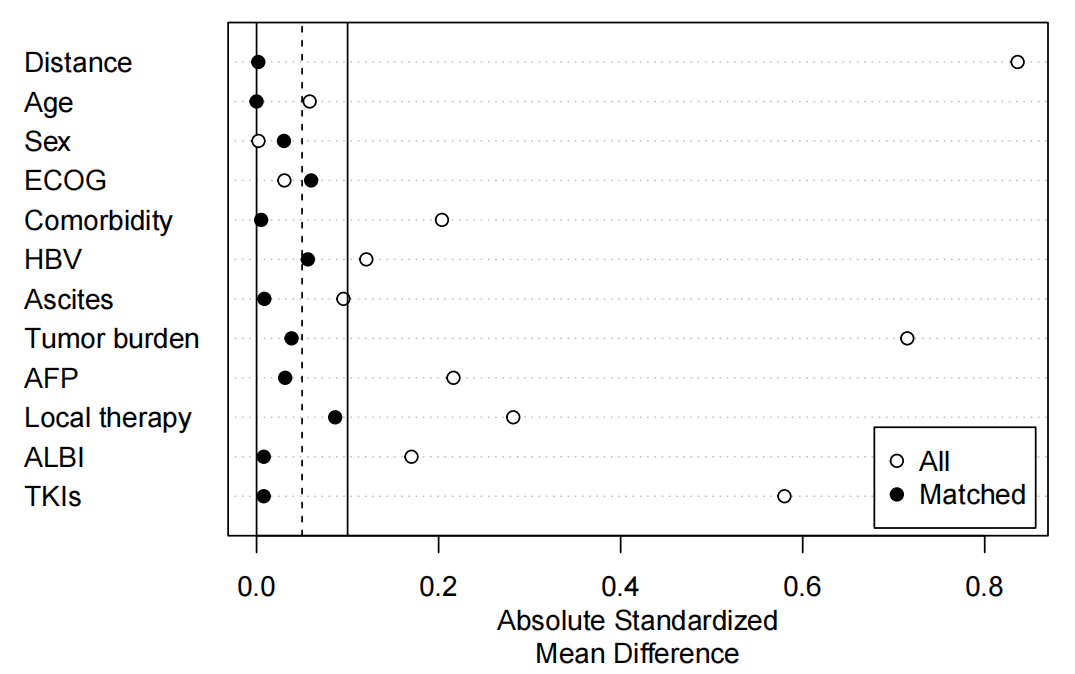


**Figure S3** Standardized differences in mean or proportion of variables before and after propensity score matching in total cohort

**Figure S4**


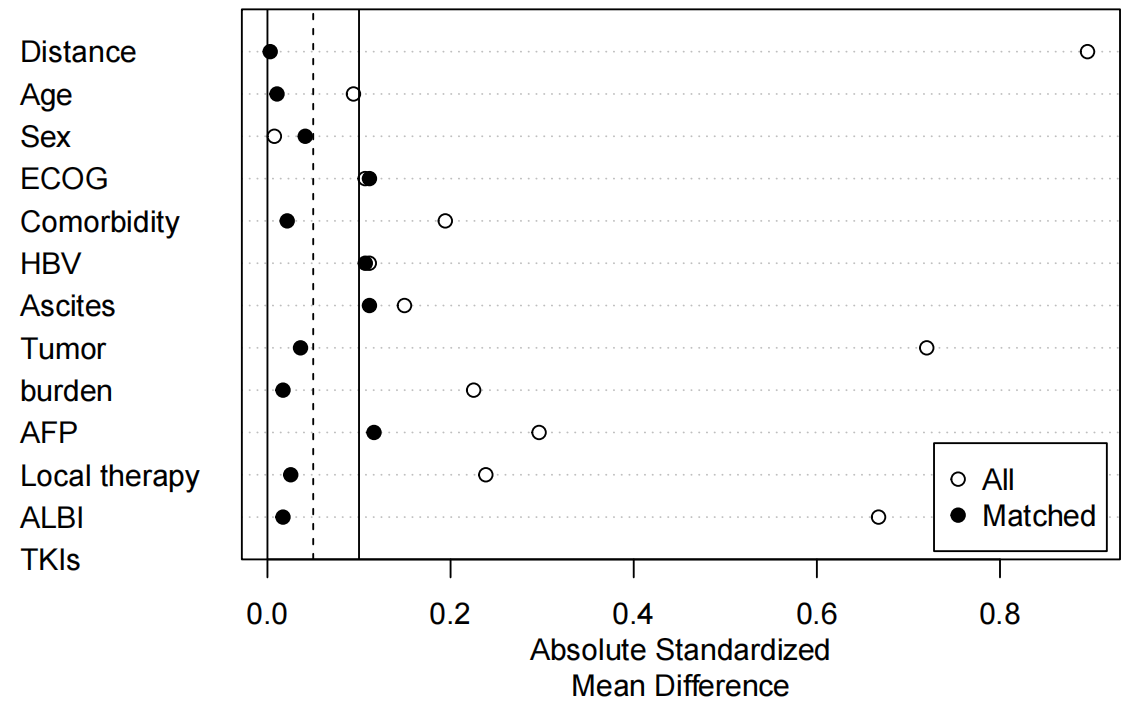


**Figure S4** Standardized differences in mean or proportion of variables before and after propensity score matching in non-nfiltrative HCC cohorts

**Figure S5**


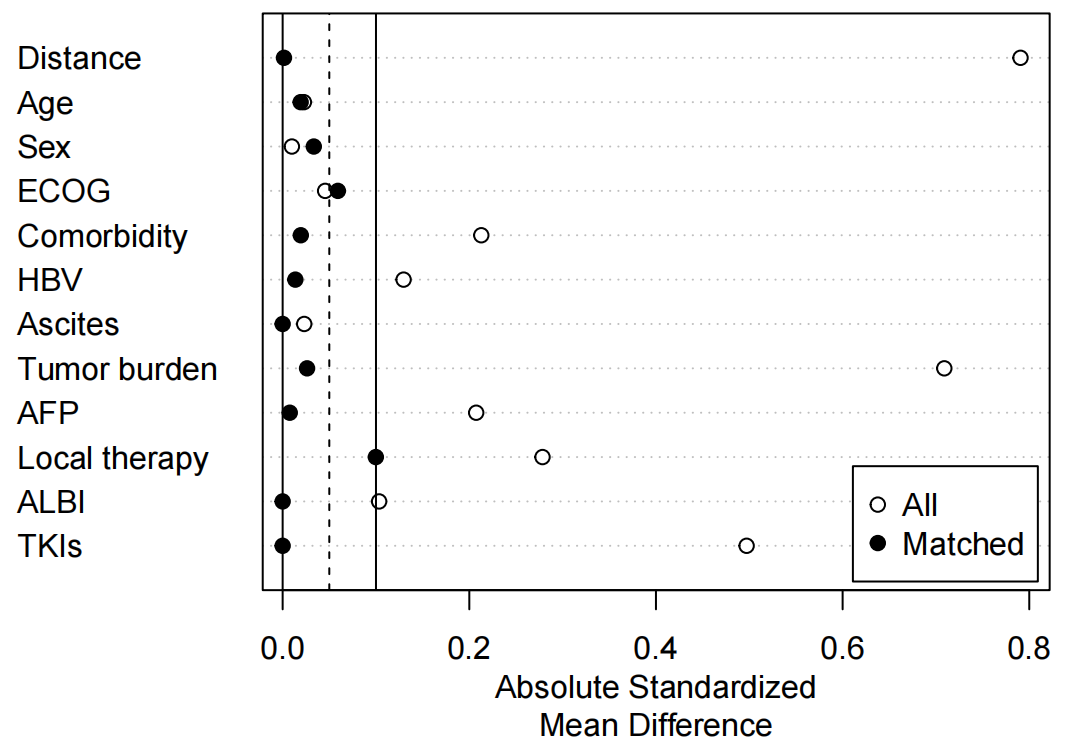


**Figure S5** Standardized differences in mean or proportion of variables before and after propensity score matching in nfiltrative HCC cohorts
